# Comparative 1D Blue-Native electrophoresis analysis of *Plasmodium falciparum* and human proteins associated with cytoadherence

**DOI:** 10.1186/s12936-018-2445-8

**Published:** 2018-08-13

**Authors:** Yang Wu, Simon C. Wagstaff, Saeed A. Al-Harthi, Alister G. Craig

**Affiliations:** 1 Liverpool School of Tropical Medicine, Pembroke Place, Liverpool, L3 5QA UK; 2 Department of Parasitology, Faculty of Medicine, Umm AL-Qura University, Makkah, Kingdom of Saudi Arabia; 3 Present Address: The Francis Crick Institute, 1 Midland Road, London, NW1 1AT UK

**Keywords:** *Plasmodium falciparum*, Cytoadherence, Blue native electrophoresis, Protein complexes

## Abstract

**Background:**

To understand more about changes to the molecular components that occur when host endothelium interacts with *Plasmodium falciparum*-infected erythrocytes, a combined technique of protein separation (1D Blue-Native electrophoresis) and mass spectrometry of infected erythrocytes with endothelial cells (EC) in a co-culture system has been used.

**Methods:**

Native proteins were extracted from co-cultures and identified by mass spectrometry. Proteomic data from different parasite strains, either adhesion proficient (to endothelial cells) or non-adherent, were analysed in parallel to reveal protein associations linked to cytoadherence. Informatic approaches were developed to facilitate this comparison.

**Results:**

Blue-Native gel separation and LC/MS/MS identification revealed major differences in samples produced from endothelial cell co-culture with adherent and non-adherent parasite strains. This approach enabled us to identify protein associations seen only with the adhesion proficient parasite strain.

**Conclusions:**

The combination of proteomic and analytical approaches has identified differences between adherent and non-adherent parasite lines in co-culture with EC, providing potential candidates for complexes or associations formed during cytoadherence involved in cell structure, signalling and apoptosis.

**Electronic supplementary material:**

The online version of this article (10.1186/s12936-018-2445-8) contains supplementary material, which is available to authorized users.

## Background

*Plasmodium falciparum* is the deadliest of the six human-infecting malaria species and responsible for the majority of malaria-related deaths. A unique characteristic of this species is its ability, in mature form infected erythrocytes (IE), to undergo a range of adhesive interactions, such as the binding of IE with endothelial cells (EC) (cytoadherence), and the interaction of IE with non-infected erythrocytes (rosetting) and with other IE (autoagglutination). The severe clinical manifestations caused by *P. falciparum* are thought to be mediated, at least in part, by sequestering IE and/or by rosetting between infected and uninfected red blood cells to form clumps in the microvasculature of major organs such as the brain, lung and kidney [1, 2]. The primary mechanism underlying sequestration of the asexual-stage *P. falciparum* IE to EC is mediated by the diverse *var* gene products, *P. falciparum* erythrocyte membrane protein-1 (PfEMP1), that are displayed on the surface of IE and can bind to several host proteins, although recently other variant surface protein families have been implicated in rosetting [3, 4].

Cytoadherence in *P. falciparum* is a complex process involving a range of host receptors interacting with the parasite-encoded proteins. Well-characterized host molecules include ICAM-1, CD36, EPCR and chondroitin sulphate-A (CSA) binding to specific PfEMP-1 domains from the parasite [5]. Although several molecules have been characterized in terms of the expression patterns, gene organization and their involvement in cytoadherence, the molecular mechanisms underlying how the parasite proteins are able to modulate the behaviour of host EC have not been solved. Many adhesion receptors have co-operative functions that contribute to strengthening cytoadherence, such as between ICAM-1 and CD36 or EPCR in mediating adherence of *P. falciparum* IE to cultured human microvascular EC [6–8].

PfEMP1 is known to interact with several proteins, both parasite and host derived, in the knob complex at the surface of the infected erythrocyte. The cytoplasmic acidic terminal segment of PfEMP1 has been shown to interact with KAHRP [9, 10] and PHIST [11, 12] proteins as well as erythrocyte cytoskeleton components, such as actin, spectrin and ankyrin. On the host side, a large number of endothelial complexes have been identified, many involved in ligand capture and signalling events, for example the angiopoietin/Tie2 system [13]. The large dynamic range in protein abundance and the complexity of both proteomes present a challenge for analysis of these interactions. This work has focused on sample preparation procedures to reduce sample complexity and potentially to increase the detection of low-abundance proteins. The first qualitative approach used metabolically-labelled IE co-cultured with non-labelled EC to visualize global changes to IE proteomes during co-culture. This was followed by identification of proteins and associated complexes from co-culture systems using Blue Native (BN) gel electrophoresis [14]. Changes in potential interacting partners formed during cytoadherence were identified by computational analysis of the BN gel fractions from spatial comparisons of proteomic ‘hits’, based on the position of the band from the binding parasite line sample compared with the corresponding band in the non-binding parasite line and the bands either side of this, to allow for slight misalignment of the two lanes on the BN gel.

## Methods

### Parasite culture

*Plasmodium falciparum* clones and isolates used in this study were C24 [15], ItG [16] and 3D7 [17]. C24 and 3D7 bind to CD36 but not ICAM-1, whereas ItG binds strongly to both receptors. Parasites were cultured in vitro in group O^+^ human erythrocytes using previously described conditions [18]. Briefly, parasites were cultured in RPMI-1640 medium (supplemented with 37.5 mM HEPES/7 mM d-glucose/6 mM NaOH/25 µg/ml gentamicin sulphate/2 mM l-glutamine/10% human serum) at a pH of 7.2 in a gas mixture of 96% nitrogen, 3% carbon dioxide and 1% oxygen. To minimize the effect of antigenic switching in culture, a batch of stabilates was prepared from a post-selection culture and used for no more than 3 weeks. IE were synchronized by 5% sorbitol treatment, the parasites used for co-culture studies were at 25–30 h after invasion; mature trophozoites were enriched by Plasmagel flotation.

### Endothelial cells and co-culture conditions

Pooled human umbilical vein endothelial cells (HUVEC) were obtained from Promocell (Heidelberg, Germany). HUVEC cultures were maintained as previously described [19]. HUVEC cells were used as they have a similar repertoire of expression of receptors as brain endothelium, in particular having very low levels of CD36 but, on stimulation with TNF, very high levels of ICAM-1. Briefly, HUVEC were grown in complete endothelial cell medium containing 2% fetal bovine serum (Promocell). Cells at passages five to six were used when they were confluent on 1% gelatin (Sigma, UK) coated flasks. For 2D gel analysis, EC were co-cultured with IE in the presence of TNF; for studies on protein complexes, TNF-activated EC were co-cultured with IE as previously described [20], with modifications. Briefly, HUVEC grown to confluence in 75 cm^2^ flasks were activated with TNF (0.5 ng/ml) for 8 h at 37 °C. Before the co-culture experiment, TNF was removed and the HUVEC were washed twice with binding buffer (RPMI-1640 with HEPES modification and 0.2% glucose, pH 7.2). An enriched (40% parasitaemia) mature-trophozoite IE suspension (25–30 h after invasion) was adjusted to 1% haematocrit in binding buffer (RPMI-1640 with HEPES modification and 0.2% glucose, pH 7.2) and applied to a mono-layer of TNF-activated HUVEC, co-cultured for 30 min with agitation every 10 min. Un-bound IE were then washed off and the bound IE and EC further extracted and analysed. All parasite and EC cultures were regularly monitored for mycoplasma using the Takara PCR mycoplasma detection kit (Clontech).

### Metabolic labelling of IE and fluorography

The metabolic labelling of parasites has been described previously [21] used here with modifications. Briefly, IE grown to trophozoite stage (25–30 h after invasion) were enriched by Plasmagel flotation and adjusted to 40% parasitaemia. The IE were washed with serum-free RPMI-1640 medium without methionine three times. In vitro metabolic labelling was carried out in methionine-free RPMI medium by the addition of 50 µCi/ml [^35^S] methionine for 4 h under standard culture conditions. The reaction was stopped by removing the radioisotope via centrifugation and washing with binding buffer. The labelled parasites were lysed with hypotonic buffer [1/10 dilution of binding buffer with 1× proteinase inhibitor (cOmplete™, Mini, Roche, Germany)], then neutralized with binding buffer (1/10, v/v), centrifuged at 3000×*g* for 10 min, the supernatant was used as the soluble protein fraction, the pellet was used as the membrane fraction. The labelled parasites were also used intact in co-culture experiments. After co-culture, protein extraction was performed as described (see below) under denaturing conditions and subjected to 2D-electrophoresis. After electrophoresis, the gel was fixed and stained with Coomassie Blue, immersed in Amplify (Amersham) for 30 min and dried for imaging.

### Protein extraction under denaturing and native conditions

After 30 min of co-culture, unbound IE were washed off from EC using binding buffer 3–5 times, monitoring binding levels under the microscope. C24 is a non-adhesive strain to HUVEC while ItG is a strong binder, due to the dependence of adhesion on ICAM-1 in this system. For binding with intact IE, 1 ml hypotonic buffer was added and incubated for 30 s. For denaturing 2D electrophoresis, the lysis of IE was stopped by gently adding 9 ml binding buffer. EC were washed once and excess liquid was removed. 2D rehydration buffer [8 M urea, 2 M thiourea, 2% CHAPS, 65 mM dithiothreitol (DTT), and 0.5% ampholyte pH 4–7 or 3–10] were added on to the EC monolayer and cells scraped and collected. IE/EC complexes are most likely to be membrane bound protein and cell-surface molecules, therefore, for native complexes analysis, lysis of IE was stopped by adding 9 ml native washing buffer (0.33 M sorbitol, 50 mM bis–tris pH 7.0), the cells were scraped, collected and washed three times. The pellet containing potential intact complexes was further extracted with detergent or diluted in protein immunoprecipitation buffer for further analysis.

### Two-dimensional electrophoresis

Samples prepared from co-culture were solubilized in 2D rehydration buffer, vortexed, sonicated on ice 10 times for 5 s followed by centrifugation at 15,000×*g* for 10 min, and the supernatant was then subjected to 2D electrophoresis. 150 µg of protein for co-culture samples was loaded. The iso-electric focusing (IEF) was performed with pre-cast immobiline Dry-strip gels using IPG-phor IEF Unit (Amersham). The running programme consists of 10 h for 30 V, 40 min for 200 V, 1 h for 500 V, 4 h for 2000 V and finally 8 h for 8000 V. The voltage was increased gradually until a total of 80,000 vh was reached. The focused strips were equilibrated in 10 ml equilibration solution (50 mM tris–HCl, pH 6.8, 6 M urea, 30% glycerol, 2% SDS) with reducing agent of 1% DTT for 10 min, and 10 ml equilibration solution with 4.5% iodoacetamide for another 10 min. The strips were then washed twice briefly with 1× SDS gel running buffer and loaded on 10% or 12.5% SDS-PAGE gels for second dimension separation. The gels were usually run overnight using a Laemmli buffer system [22].

### 1D Blue-Native (BN) gel electrophoresis

Blue-Native gels were prepared according to Schagger et al. [14] with slight modifications. The membrane fraction from co-culture experiments was collected as described above (insoluble pellet of 0.33 M sorbitol/50 mM bis–tris-HCl, pH 7.0) and extracted using 25 mM bis–tris-HCl, pH 7.0 with 2% digitonin in 20% (w/v) glycerol, for 60 min on ice with agitation. Insoluble material was removed by centrifugation at 18,000×*g* for 30 min. The soluble part was mixed in 10 to 1 ratio with BN gel sample buffer (100 mM bis–tris-HCl, pH 7.0/0.5 M 6-amino-*n*-caproic acid/30% sucrose/5% Serva Blue G) and applied to 0.75 mm thick/5–15% BN gradient gels in an Amersham Hoefer SE 600 vertical unit. Gels were run at a constant voltage of 150 V at 10 °C for approximately 8 h. The cathode buffer (50 mM tricine/15 mM bis–tris) was exchanged with buffer lacking dye after the top 1/3–1/2 of the gel was covered with dye (~ 2 h). Gels for immunoblotting were incubated in transfer buffer with 0.1% SDS for 10 min at room temperature before transferring.

### Western blot on 1D Blue-Native (BN) gel

Blue-Native gels were prepared and run as described above. The duplicated gel was incubated in transfer buffer (25 mM Tris-base, 190 mM glycine, 20% methanol) with 0.1% SDS for 10 min at room temperature, then electrophoretically transferred to nitrocellulose, and the membranes were blocked by overnight incubation in 5% skim-milk in Tris/saline/Tween (TST: 0.01 M Tris, pH 8.5, 0.15 M sodium chloride, 0.1% Tween 20). The blot was incubated with non-adhesion blocking mouse monoclonal antibody to ICAM-1 (GP89-14, gift from Prof Judy Johnson) (1:2000) and rabbit antisera to *HSP60* (1:5000) dilution in TST. The secondary antibodies goat anti-mouse or rabbit IgG (heavy + light chain) horseradish peroxidase conjugate (Nordic, 1:3000) were used to localize antibody-antigen complexes. Blots were developed using ECL Western blot detection reagent (GE Healthcare, UK).

### In gel digestion for nano-flow LC/MS/MS

A set of intact complexes from co-culture were produced by separation on BN gels and identified by mass spectrometry. Some specific bands were also identified by immunoblot using anti-ICAM-1 and HSP60 antibodies (see Additional file 1). The sub-proteomes were achieved by excising protein bands from the BN gel according to the diagram in Fig. 2. The bands were excised from the same gel, to ensure consistent running conditions, put into an Eppendorf Ultra Pure 1.5 ml centrifuge tube and then cut into 1 mm^3^ cubes and rinsed twice in 200 µl MilliQ water for 15 min. The gel slices were dehydrated by the addition of 100 µl of 50% (v/v) acetonitrile/water, incubated at room temperature for 10 min which was then removed. 100 µl of ammonium bicarbonate (50 mM) was then added to each sample and incubated again at room temperature for 10 min. These last two steps were repeated. After removal of the ammonium bicarbonate, 10 µl of Promega (Southampton, UK) sequence grade trypsin (10 µg/ml in 50 mM ammonium bicarbonate) was then added to the gel fragments and incubated at 37 °C for 18 h (overnight), after which the supernatant was removed and kept. 20 µl of 70% acetonitrile (v/v in water) was added to the gel and incubated for 30 min at room temperature. The supernatant was then removed and pooled with the previous supernatant. The combined supernatant was dried in a speed-vac and resuspended in 12 µl of 0.1% formic acid.

### Mass spectrometry and database searching

Nanoflow LC–MS/MS analysis and database searching were according to previously described methods [23, 24] using liquid chromatography separation (Ultimate 3000 LC, DIONEX) coupled to an LCQ Deca XP plus Ion-trap Mass Spectrometer (Thermo Finnigan, Palo Alto, CA USA). The samples were initially desalted and concentrated on a C18 peptide trap. The peptides were then separated on a C18 PepMap nanocolumn (3 μl 100 Å, Dionex). Following sample injection, peptides were eluted with a 50 min gradient of 0–50% acetonitrile at a flow rate of 0.3 μl/min. The mass spectrometer was operated on a data dependent ‘Triple play’ mode where the three most intense ions in the full scan were subjected to a zoom scan followed by MS/MS. MS analysis was performed on an LCQ Deca XP plus Ion-trap Mass Spectrometer using Xcalibur (version 2.1) software (Thermo Scientific, UK). Ions were scanned between 350 and 2000 *m/z* in positive polarity mode. The ion-trap operated with CID MS/MS (with wide band activation) on the 20 most intense ions.

Recent work from other groups has used combined human/parasite databases [25, 26], therefore the raw data from the BN gel slices were searched using the appropriate combinations of the Uniprot human protein sequence database (downloaded 17.7.17) combined with the appropriate *P. falciparum* (release 33, June 2017) 3D7 or ItG annotated protein sequences. Proteins were identified using Thermo Proteome Discoverer v 1.4 by combining Sequest HT and Mascot searches in a single workflow with the following parameters (all other values at default). Precursor mass tolerance 0.5 Da; Fragment mass tolerance 0.3 Da; N-terminal acetylation and oxidation of methionine as variable modifications; carbamidomethyl cysteine as fixed modifications; full trypsin digestion with max missed cleavages 2. Search engine results were combined and subjected to target decoy PSM validation operating a strict FDR significance threshold of 1%. The mass spectrometry proteomics data have been deposited to the ProteomeXchange Consortium via the PRIDE partner repository with the dataset identifier PXD009154.

### Bioinformatics

The goal for the informatics approach was to derive a list of proteins unique to bands analysed with the binding parasite line, that were not seen in a similar location (the corresponding band plus the bands either side) in the non-binding sample. Two approaches were used to compare the protein lists produced by the MS/MS analysis. Proteins IDs were manually inspected to identify matches between corresponding bands in the binding (ItG) and non-binding (3D7) samples, by comparing an ItG band to the corresponding band in the 3D7 track as well as the two adjacent 3D7 bands. This included direct matches of sequence identifiers as well as clear overlaps in the annotation (e.g. different entries for the same or very similar proteins). For the human samples this was relatively straightforward due to the depth of the annotation but for the parasite entries the lack of annotation of the IT genome was a challenge, as was how to handle members of gene families, such as PfEMP1. For the latter, any overlapping ID of PfEMP1 or EMP1 was scored as being a ‘match’ and removed from the final list, although this may subtract real differential hits for this protein.

To provide an unbiased computer approach to protein identification, the composition of each of the 32 gel bands excised from the binding isolate was compared with the equivalent band, plus adjacent bands, from the non-binding isolate before further processing to provide a non-redundant list of proteins identified in each binding isolate gel band. Briefly, accession numbers in each band from the binding isolate (B^1–32^) were compared with a merged file containing accession numbers from an equivalent overlapping window of bands from the non-binders group (comprising each equivalent band from the non-binding isolate [NB^1–32^] and two adjacent thin bands i.e. NB^+1^ and NB^−1^). In a refinement to this strategy, the output of this initial comparison (a set of protein sequences unique to each NB band), was compared (by BLASTP) to protein sequences contained within each of the merged files to further exclude related sequences on the basis of a shared level of identity [Human data: > 65% over a min 100aa overlap in almost all cases; two matches of 100% over 80aa were also accepted as identity; Parasite data: > 40% over a minimum of 100aa (the lower limit allows for variation between the ItG and 3D7 genomes)]. The final output was a non-redundant set of proteins detected in each band from the binding isolate. Further details of the technical specifications of this pipeline are available on request.

Functional analyses were carried out on the final lists for parasite and human proteins, but only the latter showed any coherent structure. Ninety-five non-redundant protein sequences identified in binding experiments were annotated by BLASTP and Interpro using Blast2GO v4.1.9 against preformatted NCBI nr protein database volume 68 (14.6.17) [default parameters except Blast E-value 1 × 10^−5^; Max hits 20] prior to mapping and annotation using default parameters. GO annotations for molecular function are displayed as a combined graph (sequence filter 15; Nodescore filter 35; Nodescore alpha 2). Decoration by Nodescore highlights significant contributions from annotations associated with binding including the formation of complexes, dimers and cytoskeletal protein binding (see Additional file 2).

## Results

**Fig. 1:**
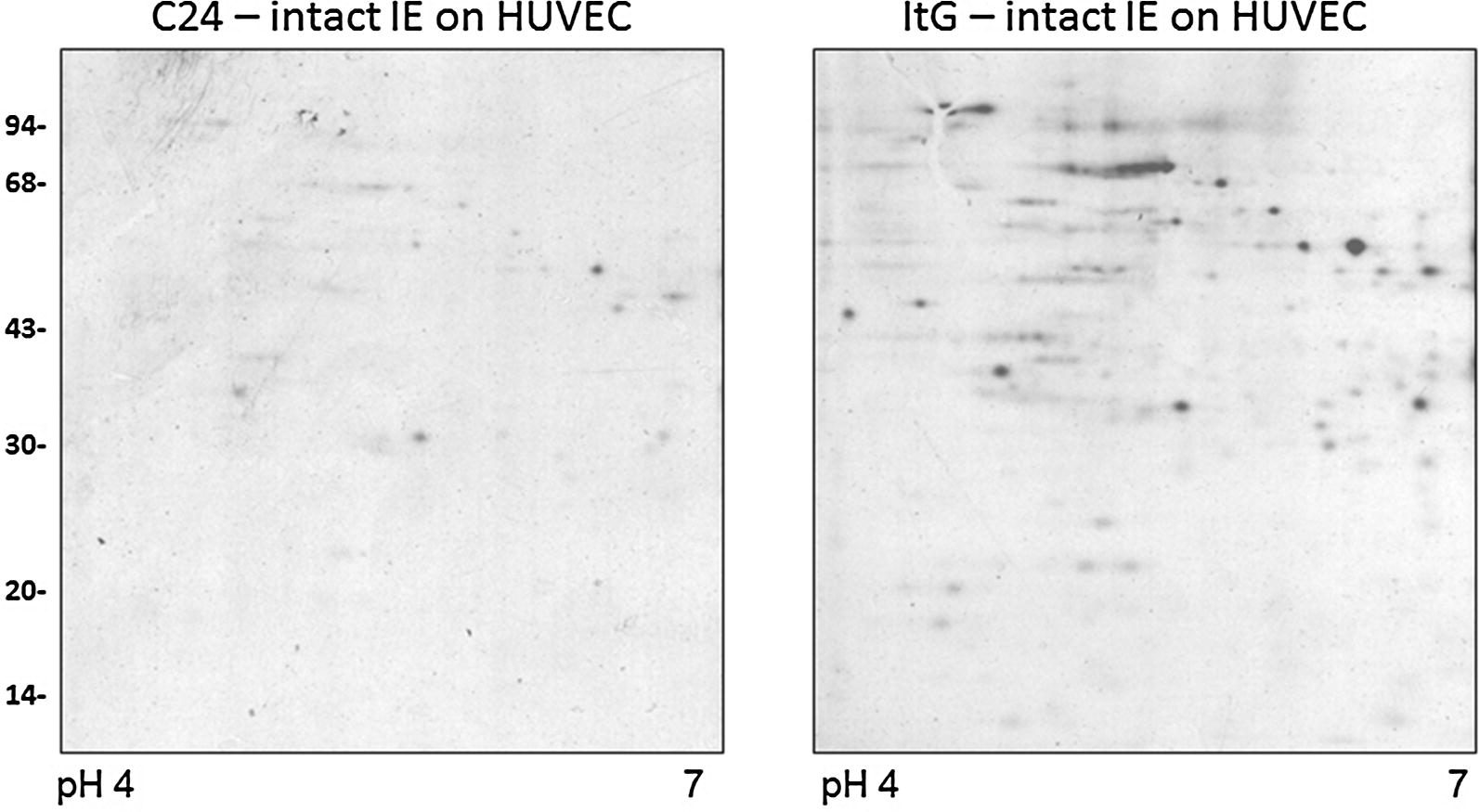
Parasite proteins associated with endothelial binding. Synchronized C24 and ItG parasites grown to mature stage (approximately 25–30 h post-invasion) were enriched by Plasmagel flotation and metabolically labelled using ^35^S methionine. The labelled IE were used intact in co-culture with confluent TNF-activated HUVEC cells for 30 min after which unbound IE were removed and proteins prepared from the adherent cells. The figure shows autoradiographs of samples run on IEF gels (pH 4–7) followed by 12.5% SDS-PAGE co-culture with intact C24 infected erythrocytes with HUVEC (left) and intact ItG infected erythrocytes with HUVEC (right). Corresponding gels stained with Coomassie blue were used to ensure equivalent loading (data not shown). Scale on left hand side in kDa

The first phase of this investigation was to examine the profiles of parasite proteins potentially involved in adhesion to endothelium. To investigate this, metabolically labelled IE were used in the co-culture system where intact trophozoites of ItG (adherent) and C24 (non-adherent) strains were exposed to HUVEC. Figure 1 shows the 2D gel IE-fraction binding profiles and while ItG shows several proteins revealed by this approach, C24 shows fewer protein spots than ItG due to the very much reduced binding to HUVEC demonstrated by this parasite line. This is in line with the binding phenotypes of the two parasite variants, as ItG is able to bind to ICAM-1, which is expressed at high levels on TNF-activated HUVEC, whereas C24 and the 3D7 line also used in this study do not bind to ICAM-1 but show adhesion to CD36, which is not expressed on HUVEC. This approach gives a picture of the potential abundance of adhesion-dependent proteins but does not provide specific information about complexes or associations as the presence of proteins could be due to being carried along in membrane components rather than directly interacting with other host or parasite molecules.

**Fig. 2:**
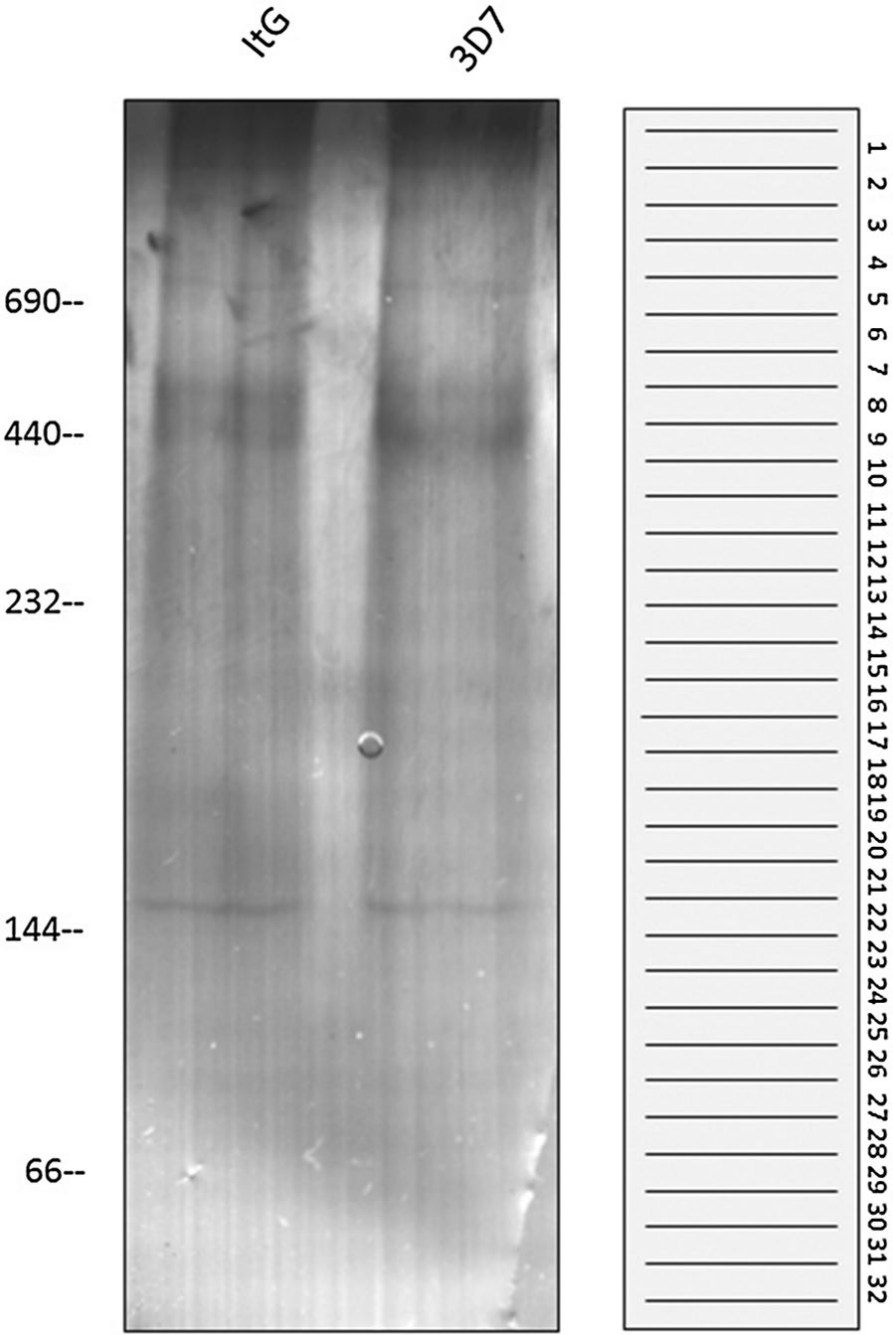
One-dimensional Blue-Native gel electrophoresis of co-cultured samples. One-dimensional Blue-Native (BN) gel electrophoresis was performed on samples prepared to retain protein complexes from co-culture of non-adherent (3D7-HUVEC) and adherent (ItG-HUVEC) parasite lines and TNF-activated HUVEC. The figure shows the Coomassie blue stained 1D BN gel and a schematic of the position of slicing bands from 1D BN gel for protein identification

To improve the identification of protein associations/complexes formed differentially under adherent and non-adherent conditions, BN gel separation coupled with mass spectrometry was used. Shown in Fig. 2 is a Coomassie blue stained BN 1D gel containing samples extracted from ItG (adherent) and 3D7 (non-adherent) IE co-cultured with HUVEC. As can be seen from this figure, the native proteins were separated well in the range from 700 to 50 kDa, and showed equal amounts of proteins loaded on each gel. These protein lanes were excised into 32 thin bands per lane (Fig. 2) and processed separately for identification by LC/MS/MS.

All proteins extracted and separated by this native protein method should represent a small pool of complexed (and uncomplexed) proteins in the given sample. Therefore, a comparison of protein pools was made using samples derived from co-culture of HUVEC with 3D7 and ItG, the expectation being that the 3D7 samples would provide ‘background’ identifications that could be subtracted from the ItG samples, which should include associations/complexes formed under adherent conditions. The MS identification data were analysed based on the position in the gel in Fig. 2 and those identified in the ItG sample were checked for the presence of identical or similar IDs in the corresponding and flanking 3D7 bands, both manually and using a computer-based approach. After subtraction based on ID matching and BLAST searches, proteins remaining (parasite and human) were considered as being in potential protein complexes that differ between binding and non-binding parasites. The full lists of identified human and parasite proteins are shown in Additional files 3 and 4, respectively and the final list post-subtraction, with parasite and host proteins shown side by side, in Table 1. Specific candidates are discussed in more detail below, but briefly from the human EC component there were a significant number of cytoskeleton proteins, some with signalling functions (ezrin; girdin; tubulin) as well as a smaller number of specific signalling molecules (tyrosine-phosphorylated regulated kinase 2). On the parasite side, PfEMP1 was only seen in the ItG samples in bands 6 and 14. Some parasite-derived signalling proteins were also seen. While the subtraction process did remove some background, some non-specific hits were retained, seen in the recovery, for example, of a number of mitochondrial and ribosomal proteins and several keratins (most likely due to contamination from human epidermis during sample preparation).

**Table 1:** Proteins identified through differential BN gel analysis

	Human		Parasite	
Band 1	P50990	T-complex protein 1 subunit theta	PFIT_0712500	Calmodulin
	P23396	40S ribosomal protein S3	PFIT_1211500	Ca2+-dependent ATPase
	Q13409	Cytoplasmic dynein 1 intermediate chain 2		
Band 2	P04264	Keratin, type II cytoskeletal 1	PFIT_0216100	Autophagy-related protein 11, putative
	P13645	Keratin, type I cytoskeletal 10	PFIT_1321500	Translation initiation factor EIF-2B subunit related
	P17661	Desmin		
	P35527	Keratin, type I cytoskeletal 9		
	A6NNL0	NUT family member 2B		
Band 3	P17661	Desmin	PFIT_1235400	ATP synthase subunit beta, mitochondrial
	P13645	Keratin, type I cytoskeletal 10	PFIT_0703500	E3 ubiquitin-protein ligase
	Q92630	Dual specificity tyrosine-phosphorylation-regulated kinase 2	PFIT_1247800	Unknown function
	Q92545	Transmembrane protein 131	PFIT_0303600	Lipoamide acyltransferase component of branched-chain alpha-keto acid dehydrogenase complex
	Q86YH2	Zinc finger protein 280B		
Band 4	P04075	Fructose-bisphosphate aldolase A	PFIT_1235400	ATP synthase subunit beta, mitochondrial
	Q14203	Dynactin subunit 1	PFIT_1403800	Unknown function—RBP homology
	P21796	Voltage-dependent anion-selective channel protein 1	PFIT_0529300	Acyl-CoA synthetase
	Q13242	Serine/arginine-rich splicing factor 9	PFIT_1032700	Unknown function
Band 5	P21796	Voltage-dependent anion-selective channel protein 1	PFIT_1415500	Atypical protein kinase, ABC-1 family, putative
	Q8IVL1	Neuron navigator 2	PFIT_1025500	Unknown function
	Q9GZL7	Ribosome biogenesis protein WDR12		
Band 6	A5A3E0	POTE ankyrin domain family member F	PFIT_0712500	Calmodulin, putative
	P40939	Trifunctional enzyme subunit alpha, mitochondrial	PFIT_0533600	Unknown function—Ser/Thr kinase/Arginase
	Q6ZSB9	Zinc finger and BTB domain-containing protein 49	PFIT_1415500	Atypical protein kinase, ABC-1 family, putative
			PFIT_1017700	Serine/threonine protein phosphatase 8, putative
			PFIT_1400200	Erythrocyte membrane protein 1, PfEMP1
Band 7	P00915	Carbonic anhydrase 1	PFIT_0514400	Aspartate–tRNA ligase, putative
	P35527	Keratin, type I cytoskeletal 9		
	Q96T23	Remodeling and spacing factor 1		
	Q008S8	Epithelial cell-transforming sequence 2 oncogene-like		
	O43506	Disintegrin and metalloproteinase domain-containing protein 20		
	Q8TE49	OTU domain-containing protein 7A		
Band 8	P00915	Carbonic anhydrase 1	PFIT_1022600	Dynein heavy chain, putative
	P04075	Fructose-bisphosphate aldolase A	PFIT_0421800	Unknown function—Arginase?
	P04264	Keratin, type II cytoskeletal 1		
	P15311	Ezrin		
Band 9	P00915	Carbonic anhydrase 1	PFIT_1006100	Unknown function
	P04075	Fructose-bisphosphate aldolase A	PFIT_0809500	Kinesin-like protein, putative
	P14406	Cytochrome c oxidase subunit 7A2, mitochondrial		
	P01138	Beta-nerve growth factor		
Band 10	P00915	Carbonic anhydrase 1	PFIT_1006100	Unknown function
	P60709	Actin, cytoplasmic 1	PFIT_0533600	Unknown function—Ser/Thr kinase/Arginase
	Q12849	G-rich sequence factor 1	PFIT_1135000	Unknown function
	Q13277	Syntaxin-3	PFIT_1365300	Unknown function—Arginase?
			PFIT_1033700	Thioredoxin-like associated protein 2, putative
Band 11	Q06830	Peroxiredoxin-1	PFIT_1364900	Ubiquitin-60S ribosomal protein L40
	P62979	Ubiquitin-40S ribosomal protein S27a	PFIT_1415500	Atypical protein kinase, ABC-1 family, putative
	P35030	Trypsin-3	PFIT_0516200	Cation-transporting ATPase 1
	Q96RS6	NudC domain-containing protein 1	PFIT_1310300	Unknown function—nuclear structural protein?
	Q3V6T2	Girdin	PFIT_1359000	Unknown function—Pf-SET1 homology
	Q15075	Early endosome antigen 1		
	Q7Z429	Protein lifeguard 1		
Band 12	P13645	Keratin, type I cytoskeletal 10	PFIT_1201800	Unknown function—protein kinase?
	P35908	Keratin, type II cytoskeletal 2 epidermal	PFIT_1022600	Dynein heavy chain, putative
	Q8TCT9	Minor histocompatibility antigen H13		
	Q13277	Syntaxin-3		
Band 13	P00338	l-lactate dehydrogenase A chain	PFIT_1222000	Endoplasmin, putative
	P32119	Peroxiredoxin-2	PFIT_0709100	Unknown function
	Q9UFH2	Dynein heavy chain 17, axonemal		
	P27824	Calnexin		
	P04264	Keratin, type II cytoskeletal 1		
Band 14	P14618	Pyruvate kinase PKM	PFIT_bin02200	A4varTres-erythrocyte membrane protein 1, putative
	Q13277	Syntaxin-3	PFIT_1033700	Thioredoxin-like associated protein 2, putative
Band 15	Q9Y4L1	Hypoxia up-regulated protein 1	PFIT_0821800	Heat shock protein 70
	P11142	Heat shock cognate 71 kDa protein	PFIT_1222000	Endoplasmin, putative
	P13645	Keratin, type I cytoskeletal 10		
	Q6UXS9	Inactive caspase-12		
	Q13277	Syntaxin-3		
	Q9HD43	Receptor-type tyrosine-protein phosphatase H		
Band 16	Q5T0J7	Testis-expressed protein 35		
Band 17	Q9Y250	Leucine zipper putative tumor suppressor 1	PFIT_0312000	E3 ubiquitin-protein ligase, putative
	P32119	Peroxiredoxin-2		
Band 18	P37802	Transgelin-2	PFIT_0821800	Heat shock protein 70
	P16520	Guanine nucleotide-binding protein G(I)/G(S)/G(T) subunit beta-3	PFIT_1015800	Glycophorin binding protein
	P61604	10 kDa heat shock protein, mitochondrial	PFIT_1364900	Ubiquitin-60S ribosomal protein L40
	P40926	Malate dehydrogenase, mitochondrial	PFIT_1424700	Unknown function—Arginase?
	Q96KP4	Cytosolic non-specific dipeptidase	PFIT_1366800	Secreted ookinete protein, putative—fibrinogen receptor homology
	P62979	Ubiquitin-40S ribosomal protein S27a		
	Q6ZU64	Cilia- and flagella-associated protein 65		
Band 19	Q99536	Synaptic vesicle membrane protein VAT-1 homolog	PFIT_0108600	Selenocysteine-specific elongation factor selB homologue, putative
	A6NCI8	Uncharacterized protein C2orf78	PFIT_1108600	Transcription factor with AP2 domain(s)
	P30485	HLA class I histocompatibility antigen, B-47 alpha chain		
Band 20	P38646	Stress-70 protein, mitochondrial	PFIT_1324100	l-lactate dehydrogenase
	P35527	Keratin, type I cytoskeletal 9	PFIT_1364900	Ubiquitin-60S ribosomal protein L40
	Q9HDC9	Adipocyte plasma membrane-associated protein	PFIT_1246400	Actin I
	Q8IXQ4	GPALPP motifs-containing protein 1	PFIT_0821800	Heat shock protein 70
	Q8TF05	Serine/threonine-protein phosphatase 4 regulatory subunit 1	PFIT_1356100	Elongation factor 1-alpha
	Q9Y3P9	Rab GTPase-activating protein 1	PFIT_1416700	Serine C-palmitoyltransferase, putative
	Q9H0V9	VIP36-like protein	PFIT_0930800	Peptidyl-prolyl cis–trans isomerase
	Q9NR99	Matrix-remodeling-associated protein 5	PFIT_0304700	Unknown function
Band 21	Q15084	Protein disulfide-isomerase A6	PFIT_0834600	Heat shock protein 70
	P38646	Stress-70 protein, mitochondrial	PFIT_1324100	l-lactate dehydrogenase
	Q96PU4	E3 ubiquitin-protein ligase UHRF2		
	P11465	Pregnancy-specific beta-1-glycoprotein 2		
	Q9H6F5	Coiled-coil domain-containing protein 86		
Band 22	Q9HDC9	Adipocyte plasma membrane-associated protein	PFIT_0707300	Heat shock protein 90
	Q8TF05	Serine/threonine-protein phosphatase 4 regulatory subunit 1	PFIT_0533600	Unknown function—Ser/Thr kinase? Arginase?
	Q9Y6N3	Calcium-activated chloride channel regulator family member 3	PFIT_0821800	Heat shock protein 70
	Q6ZTB9	Putative zinc finger protein 833	PFIT_0910700	DNA repair protein REV1, putative
			PFIT_1246400	Actin I
Band 23	P30101	Protein disulfide-isomerase A3	PFIT_1246400	Actin I
	P32119	Peroxiredoxin-2	PFIT_0913400	Unknown function—histone modification?
			PFIT_1206600	Unknown function
			PFIT_0513300	Unknown function—RBP homology/histone modification?
			PFIT_0410400	Unknown function—Ser/Thr kinase?
Band 24	P51148	Ras-related protein Rab-5C	PFIT_0617200	60S ribosomal protein L27a, putative
	Q9P2D7	Dynein heavy chain 1, axonemal	PFIT_0902700	Serine/threonine protein kinase, FIKK family
	Q9UBM7	7-dehydrocholesterol reductase	PFIT_1359000	Unknown function—Pf-SET1 homology
	Q8TF05	Serine/threonine-protein phosphatase 4 regulatory subunit 1	PFIT_1252300	Rhoptry neck protein 3
	Q96PU4	E3 ubiquitin-protein ligase UHRF2		
	P02750	Leucine-rich alpha-2-glycoprotein		
Band 25	P35030	Trypsin-3	PFIT_1356100	Elongation factor 1-alpha
	O95057	GTP-binding protein Di-Ras1	PFIT_0821800	Heat shock protein 70
			PFIT_1017700	Serine/threonine protein phosphatase 8, putative
			PFIT_1246900	Unknown function—Ser/Thr kinase?
			PFIT_0700800	Unknown function—Ser/Thr kinase?
Band 26	P35527	Keratin, type I cytoskeletal 9		
	O43852	Calumenin		
	P35030	Trypsin-3		
Band 27	P27797	Calreticulin	PFIT_1319900	Unknown function—Ser/Thr kinase?
	Q6NXR4	TELO2-interacting protein 2		
	O43572	A-kinase anchor protein 10, mitochondrial		
	O95696	Bromodomain-containing protein 1		
	Q8IWY8	Zinc finger and SCAN domain-containing protein 29		
	P55786	Puromycin-sensitive aminopeptidase		
	Q8TF05	Serine/threonine-protein phosphatase 4 regulatory subunit 1		
Band 28	P68371	Tubulin beta-4B chain	PFIT_1246400	Actin I
	P27797	Calreticulin	PFIT_1008200	Tubulin beta chain
	O95057	GTP-binding protein Di-Ras1	PFIT_0533600	Unknown function—Ser/Thr kinase/Arginase?
			PFIT_1469200	Unknown function – Ser/Thr kinase?
			PFIT_0317700	CPSF (cleavage and polyadenylation specific factor), subunit A, putative
			PFIT_0709100	Unknown function
			PFIT_1350900	Inner membrane complex protein 1f, putative
Band 29	Q8IUR0	Trafficking protein particle complex subunit 5	PFIT_0930800	peptidyl-prolyl cis–trans isomerase
	P68371	Tubulin beta-4B chain	PFIT_0628900	CPW-WPC family protein
	Q66K74	Microtubule-associated protein 1S	PFIT_1475300	Unknown function
	Q9H013	Disintegrin and metalloproteinase domain-containing protein 19	PFIT_1246400	Actin I
Band 30	P37802	Transgelin-2	PFIT_1246400	Actin I
	P09417	Dihydropteridine reductase	PFIT_1133900	Unknown function
	P17568	NADH dehydrogenase [ubiquinone] 1 beta subcomplex subunit 7	PFIT_1350900	Inner membrane complex protein 1f, putative
	P19827	Inter-alpha-trypsin inhibitor heavy chain H1	PFIT_1329100	Unknown function—Tyr kinase/Ser/Thr kinase?
Band 31			PFIT_0621900	Chorismate synthase
Band 32	Q13698	Voltage-dependent L-type calcium channel subunit alpha-1S	PFIT_0821100	14-3-3 protein
	Q8NBS9	Thioredoxin domain-containing protein 5	PFIT_1123800	Dynein heavy chain, putative
			PFIT_1229500	Myosin D

Summary table showing differentially identified proteins between binding and non-binding parasite lines from the BN gel slices for human and parasite (IT) components. The sequence identifier and a short description are given for each protein

To understand more about the potential complex formed around the ICAM-1/PfEMP1 interaction, immunoblot following native gel electrophoresis was performed using a non-adhesion blocking monoclonal antibody to human ICAM-1. The native ICAM-1-recognized region migrated to the position of approximately 400 kDa in the adhesion proficient ItG sample (Additional file 1: Figure S1), which ran only slightly higher in its migration than in the non-binding 3D7 sample. The denatured form of ICAM-1 in SDS-PAGE migrates at approximately 90–95 kDa depending on the level of glycosylation.

The position of the ICAM-1 complexes by immunoblot was similar to the bands identified in the BN-gel for 3D7- and ItG-HUVEC (bands 13–15) samples (Additional file 3: Table S1). The lack of identification of ICAM-1 in the post-filter human samples in the comparative BN gel analysis described above (Table 1) is probably due to the relatively close co-migration seen on BN gels and suggests that changes in this complex would not be picked up with this system, however, other cytoadherence-linked protein associations may still be identified.

Interestingly PfEMP1 was identified only in the ItG-HUVEC co-culture sample and not the non-adherent (3D7) sample, at bands 6 and 14 in the BN gel, with the higher molecular weight band potentially representing an adhesion-specific PfEMP1 complex. However, neither of these PfEMP1 matched the expected major variant for ItG (ITvar16).

Combined human and parasite databases have been used often for global searches using complex mixtures of proteins. Even though this work uses relatively restricted protein pools, this approach was taken using the data generated from the BN gel slices. Key to the differential analysis of the biological samples described in this paper is retaining their positional information in the BN gel, as alterations in migration could indicate differential complex formation, whereas a search using data combined from all gel slices from the 3D7-HUVEC and ItG-HUVEC samples would not incorporate this property.

## Discussion

An approach employing a combination of 1D BN gel electrophoresis combined with MS/MS analysis was used to identify potential candidates for differential protein complex formation. The approach relies on using a comparison of samples differing in key biological features (in this case cytoadherence to endothelium by comparing adherent and non-adherent parasite lines) followed by procedures to identify differences between the samples. An automated computational approach was validated by comparing it to manual curation of the findings, with a concordance of 92.5% based on 348 out of 376 concordant bands analysed using the human dataset. This information is shown in Additional file 3: Table S1, in which proteins remaining after manual curation have been left unshaded, rather than in light or dark blue, and computer generated unique hits are highlighted in yellow (on the sequence identifier). The concurrence of an unshaded protein name with a yellow highlighted sequence identifier or a shaded protein name with a non-highlighted identifier indicates agreement between the manual and automated methods of subtraction. The comparative approach removes a significant amount of background, although clearly several ‘contaminants’ remain and are retained in the differentially recognised protein set for completeness. This is a relatively simple approach for candidate identification that would be relevant for a wide range of biological questions where standard global subtractive approaches may not reveal differential complex formation.

A finding in this work is that actin and actin-binding proteins were abundantly located in many complexes in both the human and parasite fractions. Given the membrane source of the samples this is not a surprise, however the differential distribution of this class of proteins in the adherent and non-adherent samples suggests that cytoadherence alters the cytoskeleton/microtubule network. For example, the parasite actin-1 is seen extensively in the post-subtraction list for parasite proteins in ItG-HUVEC (see Additional files 1, 2, 3 and 4 for full protein lists). An aspect of the actin-containing cytoskeleton is that in human systems changes in this structure can be harnessed to provide a platform for signaling. For example, ICAM-1 dependent signaling requires the short cytoplasmic domain of ICAM-1 interacting with F-actin, such that the actin cytoskeleton provides the signal transduction framework. In other systems ICAM-1 associates with the actin-containing cytoskeleton and this interaction leads to modification of the cell surface distribution of ICAM-1 [27]. Previous work has also shown that PfEMP-1 expression varies between parasite lines, which results in changes in their avidity for EC receptors and induces, for example, different levels of ICAM-1 dependent signalling in the EC they bind to [20].

Apoptosis is postulated to be a way in which cytoadherence can contribute to causing disease through reduction in EC barrier integrity. The binding of IE to brain EC has been reported to induce endothelial cell apoptosis, for example, Pino et al. [28] have demonstrated IE modulation of the expression of endothelial apoptosis-related genes. Toure et al. subsequently showed that some clinical isolates could induce EC apoptosis [29] and the presence of apoptotic cells might upregulate the expression of cellular adhesion molecules, resulting in hyper-adhesiveness, leading to a greater accumulation of IE and subsequent increase in EC apoptosis [30]. In this study, identified proteins were also identified that are implicated in apoptosis such as Transgelin-2 and Thioredoxin domain-containing protein 5. Transgelin-2 is an actin-binding protein expressed in endothelial cells responsible partly for maintenance of vascular permeability [31] and associated with modulation of apoptosis [32, 33]. Variation in the induction of apoptosis by malaria parasites has been reported previously [29, 34] and it is possible that these findings are related to this potentially pathological or protective phenotype, with the latter vascular protective behaviour being associated with the differential identification of the inhibitor of apoptosis, thioredoxin domain-containing protein 5 [35].

Several groups have identified signalling pathways that are activated in host endothelium by cytoadherence, and this is reflected in the identification of classical kinases such as dual specificity tyrosine-phosphorylation-regulated kinase 2, as well as structural signalling molecules such as ezrin that act via the actin cytoskeleton and may be involved in ICAM-1 induced signalling [36]. The malaria parasite has an extensive signalling family of proteins [37] and has recently been shown to be able to respond to external stimuli [38, 39], although not yet to cytoadherence. Several signalling proteins were identified in this study from different kinase groups (e.g. FIKK) that could be taken forward as candidates for adhesion-related signalling in the parasite, linked to phenotypes such as antigenic switching and gametocytogenesis. Of interest is a putative parasite serine/threonine protein phosphatase eight member, co-localised with PfEMP1 (band 6), which belongs to a family of phosphatases involved in external cellular communication via regulating exocytosis [40], and the presence of the host cytoskeletal associated intracellular signalling molecule POTE ankyrin domain family F also in band 6, which may indicate a potential pathway for PfEMP1-mediated adhesion to influence the behaviour of the adhered endothelial cell.

An interesting new finding was the presence of Girdin in the ItG sample (band 11). The Girdin family of signalling proteins have been associated with Akt and, potentially more interesting in this context, the Wnt pathway [41]. The latter signalling pathway is involved in controlling endothelial barrier function and has been implicated recently in malaria parasite/endothelium co-culture studies [42]. Several other candidates have potential matches to pathways that may be implicated in endothelial processes linked to malaria pathology such as Peroxiredoxin-2 (anti-oxidant), Syntaxin-3 (vesicle formation), Rab GTPase-activating protein (regulation of exocytosis), Trypsin-3 (clotting cascade), Calreticulin (stimulates NO production) and ADAM19 (cell adhesion and signal transduction).

## Conclusions

In summary, an approach was used for the discovery of proteins involved in complexes to identify those potentially associated with malaria cytoadherence. Further work will be needed to confirm their participation in specific complexes and the minimal peptide identification (n = 1) seen with several samples suggests that the findings need to be treated with caution, but many of the biological roles for these candidates are consistent with phenotypes associated with adhesion-linked changes in malaria infection.

## Additional files

**Additional file 1.** One-dimensional blue-native gel electrophoresis combined with Immuno-blot analysis of co-culture complexes. Bis-tris-insoluble, Digitonin extracted co-culture proteins were separated by 1D BN-PAGE, transferred to nitrocellulose membranes and probed with antibody to human ICAM-1 and HSP60 (control). Sample lanes: 1 - co-culture of HUVEC with uninfected erythrocytes; 2 - co-culture of HUVEC with 3D7 infected erythrocytes; 3 - co-culture of HUVEC with ItG-infected erythrocytes.

**Additional file 2.** Combined graph of human proteins annotated by GO molecular function terms. Non-redundant protein sequences (30) identified in binding experiments were BLAST and Interpro annotated using Blast2GO v4.0.7 against preformatted NCBI nr database volume 41 (21.11.15) [default parameters except Blast E-value 1×10^−5^; Max hits 20] prior to mapping and annotation using default parameters. GO annotations for molecular function are displayed as a combined graph (sequence filter 15; Nodescore filter 35; Nodescore alpha 2). Decoration by Nodescore highlights significant contributions from annotations associated with binding including the formation of complexes, dimers and cytoskeletal protein binding.

**Additional file 3.** List of human proteins identified in each band for the 3D7-HUVEC and ItG-HUVEC samples. Dark and light blue shaded IDs in the ItG column show common bands also seen in relevant 3D7 bands removed by manual curation. Yellow highlighted sequence identifiers represent bands identified by the computer pipeline as being unique to the ItG-HUVEC sample when compared with the corresponding band and one band either side. Numbers of peptides identified for each protein are indicated.

**Additional file 4.** List of parasite proteins identified in each band for the 3D7-HUVEC and ItG-HUVEC samples. Dark and light blue shaded IDs in the ItG column show common bands also seen in relevant 3D7 bands removed by manual curation. Yellow highlighted sequence identifiers represent bands identified by the computer pipeline as being unique to the ItG-HUVEC sample when compared with the corresponding band and one band either side. Numbers of peptides identified for each protein (often only a single peptide) are indicated, as are potential functions for IT proteins (where available). The specific peptides for the PfEMP1 hits are indicated in green.
