# Supplementary figures and images for: Comparative 1D Blue-Native electrophoresis analysis of Plasmodium falciparum and human proteins associated with cytoadherence
Source: Malar J. 2018 Aug 13;17:293. doi: 10.1186/s12936-018-2445-8 (PMC6090645; doi:10.1186/s12936-018-2445-8)

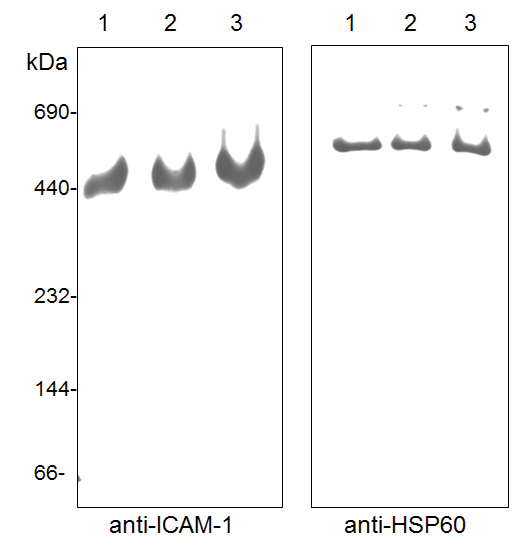

Supplement: Supplementary file 1 — Additional file 1. One-dimensional blue-native gel electrophoresis combined with Immuno-blot analysis of co-culture complexes. Bis-tris-insoluble, Digitonin extracted co-culture proteins were separated by 1D BN-PAGE, transferred to nitrocellulose membranes and probed with antibody to human ICAM-1 and HSP60 (control). Sample lanes: 1 - co-culture of HUVEC with uninfected erythrocytes; 2 - co-culture of HUVEC with 3D7 infected erythrocytes; 3 - co-culture of HUVEC with ItG-infected erythrocytes. [file 12936_2018_2445_MOESM1_ESM.tif]

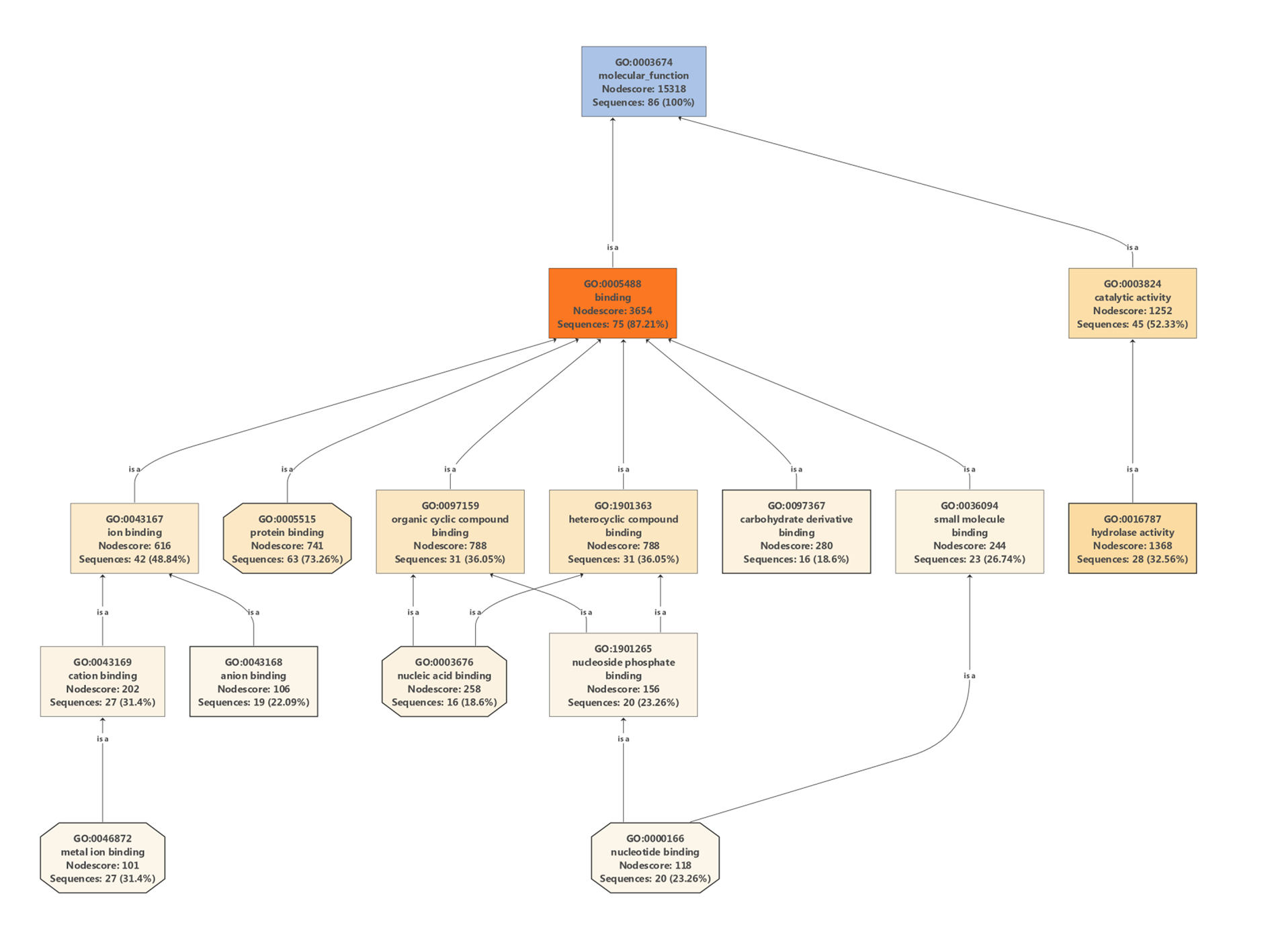

Supplement: Supplementary file 2 — Additional file 2. Combined graph of human proteins annotated by GO molecular function terms. Non-redundant protein sequences (30) identified in binding experiments were BLAST and Interpro annotated using Blast2GO v4.0.7 against preformatted NCBI nr database volume 41 (21.11.15) [default parameters except Blast E-value 1×10^−5^; Max hits 20] prior to mapping and annotation using default parameters. GO annotations for molecular function are displayed as a combined graph (sequence filter 15; Nodescore filter 35; Nodescore alpha 2). Decoration by Nodescore highlights significant contributions from annotations associated with binding including the formation of complexes, dimers and cytoskeletal protein binding. [file 12936_2018_2445_MOESM2_ESM.tif]
